# Association between clustering of unhealthy behaviors and depressive symptom among adolescents in Taiwan: A nationwide cross-sectional survey

**DOI:** 10.3389/fpubh.2023.1049836

**Published:** 2023-03-09

**Authors:** Chung Bui, Li-Yin Lin, Chun-Ji Lin, Ya-Wen Chiu, Hung-Yi Chiou

**Affiliations:** ^1^Ph.D. Program in Global Health and Health Security, College of Public Health, Taipei Medical University, Taipei City, Taiwan; ^2^Department of Health Communication and Education, Quang Ninh Provincial Center for Disease Control (CDC), Ha Long, Vietnam; ^3^Department of Leisure Industry and Health Promotion, National Taipei University of Nursing and Health Sciences, Taipei City, Taiwan; ^4^School of Public Health, College of Public Health, Taipei Medical University, Taipei City, Taiwan; ^5^Institute of Population Health Sciences, National Health Research Institutes, Miaoli County, Taiwan; ^6^Department of Public Health, Kaohsiung Medical University, Kaohsiung, Taiwan

**Keywords:** adolescents, physical activity, clustering of unhealthy behaviors, sedentary lifestyle, depressive symptoms

## Abstract

**Background:**

Among Taiwanese adolescents, how the clustering of unhealthy behaviors, including insufficient physical activity, screen-based sedentary behavior and frequent sugar-sweetened beverage consumption affecting depressive symptom remains unclear. This study aims to examine the cross-sectional association between clustering of unhealthy behaviors and depressive symptom.

**Methods:**

We analyzed 18,509 participants from the baseline survey of the Taiwan Adolescent to Adult Longitudinal Survey in 2015. The outcome was depressive symptoms, and the main exposures were insufficient physical activity, screen-based sedentary behaviors and frequent sugar-sweetened beverage consumption. Generalized linear mixed models were performed to find key factor associated with depressive symptom.

**Results:**

Depressive symptoms were common among participants (31.4%), particularly in female and older adolescents. After adjustments for covariates including sex, school type, other lifestyle factors and social determinants, individuals exhibiting clustering of unhealthy behaviors were more likely (aOR = 1.53, 95% CI: 1.48–1.58) to exhibit depressive symptoms than those who have no or only one unhealthy behavior.

**Conclusions:**

Clustering of unhealthy behaviors is positively associated with depressive symptom among Taiwanese adolescents. The findings highlight the importance of strengthening public health interventions to improve physical activity and decrease sedentary behaviors.

## Background

According to the World Health Organization (WHO), the term adolescents were defined as individuals aged between 10 and 19 years old. Adolescence basically includes two periods i.e., young adolescent (aged between 10 and 14 years) and older adolescent (aged between 15 and 19 years) (1). Adolescence is referred to the life period between childhood and adulthood, which is an important stage for human development (i.e., rapid physical growth, sexual maturation, emotional, social, and cognitive development). It is also an essential period to ensure good health status (2). Among the WHO guidelines for the health services and interventions for adolescents, mental health is certainly one of the key components to focus on. Globally, suicide ranks third among the causes of mortality in the during adolescence, whereas depression can lead to disability. Nearly half of the mental health problems start occurring by the age of 14, however, a majority of mental disorders are neglected and untreated, which can lead to serious consequences in the long-term (3).

Depression is a major risk factor for suicide, which is the second-to-third leading cause of death among adolescents (4). Depression can also lead to social, educational, and behavioral impairments such as low self-esteem, irritability, smoking, substance misuse, disordered eating (5–7). Depression is often being missed if the primary symptoms are unexplained physical symptoms, anxiety, skipping school, decline in academic participation, eating disorders, substance misuse, or behavioral problems (8). The pathogenesis of depression is complicated due to the diverse causes of this illness. For example, inherited factors, hormonal changes, familial and genetic factors, environmental factors, and brain and neuroendocrine functioning are all critical factors contributing to the incidence of depression (9–11). Similar to many health disorders, usually more than one risk factors interact and contribute the development of depression (8). These risk behaviors may frequently co-exist in a complex pathway (12). Previous studies reveal the association between depressive symptoms with overconsumption of sugar-sweetened drinks (13), inadequate physical activity (14) and sedentary behaviors (15). Meanwhile, feeling sad and hopeless are the most significant indicators for depression. Another key indicator of depression is low self-esteem, where he or she may feel foolish, unattractive, or worthless (16). In general, health risk behaviors frequently occur together to contribute to increased risk of chronic diseases and premature mortality (17). Multiple unhealthy behaviors are often more detrimental to one's health than a single behavior (18). Two studies have examined the clustering of health risk behaviors and mental health status among college samples: one from the UK (19) and one from Canada (20) revealed that students with greater numbers of unhealthy behaviors had increased risks for depression and anxiety as well as greater self-perceived psychological stress (19–21). At present, research on clustered behaviors associated with depressive symptoms remains limited.

In Taiwan, a recent Taiwan's National Epidemiological Study of Child Mental Disorders showed that the weighted lifetime prevalence rate of overall mental disorders was 31.6% among children and young adolescents. The lifetime prevalence rates of suicide-related problems: 8.2% suicidal ideation, 3.6% suicidal plans, and 0.7% suicidal attempts (22). Other previous study also found a reciprocal relationship between unhealthy eating behaviors and depressive symptoms from childhood to adolescence (23). A quasi-experimental study demonstrated the effectiveness of a 12-week brisk walking program on improving self-esteem, anxiety, and depression (24). Another large-scale project called the Health of Adolescents in Southern Taiwan examined the relationship between depression and self-esteem and social determinants in 2004 (25, 26). To date, there has been limited study investigating the co-occurrence of clustering of unhealthy behaviors among adolescents in Taiwan.

In this study, we hypothesized that the clustering of unhealthy behaviors, such as insufficient physical activity, screen-based sedentary behaviors, and frequent consumption of sugar-sweetened beverages, is positively associated with depressive symptoms. After adjusting for other factors such as social determinants and other behavioral factors, each cluster's individual associated with depressive symptoms would be assessed. As a result, this study aims to analyze the association between the clustering of unhealthy behaviors and depressive symptoms and to identify key risk factors for future intervention suggestions.

## Methods

This study followed the Strengthening the Reporting of Observational studies in Epidemiology (STROBE) guidelines (27) (Supplementary File 1).

### Sample and study design

The study is a cross-sectional secondary analysis use data from the baseline survey of Taiwan Adolescent to Adult Longitudinal Study (TAALS) in 2015. TAALS is a longitudinal nation-wide study which employed a multistage, stratified area, probability sampling design to recruit participants from both junior high schools and senior high schools in Taiwan. The methodology of the study has been described in details in another study conducted by Chien et al. (28). There were three levels of the data structure including geographic area, township-level unit, and school. The final sample contained 173 schools in 19 county/city in North, Central, East, and South Taiwan. Researchers selected 6,903 seventh-grade students (aged 13 years) in junior schools and 11,742 tenth-grade students (aged 16 years) in senior high schools, and vocational high schools. Students who failed to provide the informed consent form with their parents' signatures were excluded from the survey. For this study, data collected from those 18,509 participants who responded to all five depressive symptoms questions were used for analysis. During an interview, information was collected on sociodemographic factors, lifestyle and physical activity, substance use, dietary behaviors, mental health status, violence-related behaviors and experiences, sexual behaviors and attitudes, and social support. Ethics approval was obtained from the Joint Institutional Review Board of Taipei Medical University, Taiwan (TMU-JIRB-201410043) (28).

### Measures

#### Depressive symptoms

Information regarding depressive symptoms was collected during an on-site, self-administered survey assisted with a class-based interview system. From the Chinese version of the Center for Epidemiological Studies Short Depression Scale (CES-D) (28, 29), five questions used to investigate depressive symptoms during the past seven days were: (1) I did not feel like eating; my appetite was poor; (2) I had trouble keeping my mind on what I was doing; (3) I felt depressed; (4) I felt that everything I did was an effort.; (5) I felt lonely. The five-item scale had a high internal consistency with a Cronbach's α of 0.76. Each item was rated on a 4-point Likert-type scale ranging from 0: Rarely or none of the time (< 1 day); 1: Some or a little of the time (1–2 days); 2: Occasionally or a moderate amount of time (3–4 days); 3: All of the time (5–7 days). The sum of five responses ranged from 0 to 15. Participants who obtained a score ≥ 5 were considered having depressive symptoms (29, 30).

#### Definitions on clustering of unhealthy behaviors

Clustering of unhealthy behaviors was dichotomized by the co-existence of two or more factors including frequent sugar-sweetened beverage consumption (FSSBC), insufficient physical activity (IPA), and screen-based sedentary behaviors (SBSB) (12, 31). The reference group was participants with no unhealthy behavior or with just one unhealthy behavior. In addition, a categorical variable was generated to identify eight groups based on the amount and co-occurrence of unhealthy behaviors from no unhealthy behavior to all unhealthy behaviors (Supplementary File 2).

#### Insufficient physical activity

Similar to the Global School-based Student Health Survey (32, 33), the question “During the past seven days, how many days were you physically active for a total of at least 60 minutes per day?” was used to determine physical activity. Insufficient physical activity was defined as participants who were not physically active for a total of at least 60 min per day on five or more days during the past 7 days (34).

#### Screen-based sedentary behaviors

The question “How long have you spent watching TV, playing video games, using a computer, and playing with your mobile phone on average per day during the past seven days?” measured SBSB. A cutoff value of 2 h or more per day indicates the presence of SBSB as “yes” (≥ 2 h per day) and “no” (<2 h per day) (35).

#### Frequent sugar-sweetened beverage consumption

The question “How many times did you drink sugar-sweetened beverages (i.e., bubble milk tea, fruit juice drinks, soft drinks, sports drinks, sugary tea drinks, Yakult) during the past seven days?” indicated FSSBC. We adopted a cutoff value of three times or more to classify the frequency of consumption as “high” (≥3 times per week) and “low” (<3 times per week) (36).

#### Controlling variables

Sex, school type, body mass index (BMI), personal behaviors, school support, peer support and parental education were controlled in the predicted models of depressive symptom. We recorded sex and school type from the sampling database. Participants' weight (kg) and height (cm) were retrieved directly from school-based health record and Body Mass Index (BMI) was calculated. The Taiwan Ministry of Health and Welfare classifies BMI status as “underweight” (BMI < 18.5), “normal” (18.5 ≥ BMI < 24), and “overweight or obese” (BMI ≥ 24) (37) according to a WHO Expert Consultation for Appropriate Body-Mass Index for Asian Populations (38). Personal behaviors integrated into regression models were eating behaviors, smoking, and binge drinking. The frequency of emotional eating, skipping breakfast, eating while performing other activities, and reading nutrition labels was being divided into two categories: “low” (0–2 times/week) and “high” (3 times/week) depending on how many times the participant reported these actions during the past week. Cigarette smoking was identified (yes/no) based on self-reported frequency during the last month (33). We applied the criterion of 5 drinks on one occasion for at least 1 day during the past month to define binge drinking behavior in adolescents (39).

School support was determined by six questions including: “My teachers respect me,” “My teachers are fair,” “Teachers in my school are nice people,” “When students break rules at my school, they are treated fairly,” “My school is a good place to be,” “My school is important to me.” The six-item scale had a high internal consistency with a Cronbach's α of 0.88. Each item was rated on a 4-point Likert-type scale ranging from 1 (strongly disagree) to 4 (strongly agree). The sum of six responses ranged from 6 to 24. Participants who obtained a score ≥ 12 were considered to have school support. Four questions used to measure peer support were: “My classmate/friend really care about what happens to me,” “My classmate/friend are there for me whenever I need help,” “My classmate/friend can be trusted a lot,” “My classmate/friend care about my feelings” (a Cronbach's α of 0.90). Each item was rated on a 4-point Likert-type scale ranging from 1 (strongly disagree) to 4 (strongly agree). The sum of four responses ranged from 4 to 16. Participants who obtained a score ≥ 8 were considered to have good peer support. Being bullied experiences were investigated by “I was pushed, shoved, slapped, or kicked by other students,” “I was teased by other students,” “I was ignored or felt left out of activities or games on purpose,” and “Some pictures or words were posted online, (through email, computer text message, or Facebook), by someone else to make others laugh.” Each question was rated on a 5-point scale ranging from 1 (Never) to 5 being (Always). The sum of four responses ranged from 4 to 20. Participants who obtained a score ≥ 5 were considered to have being bullied experiences (40). Representative socio-economic factors were the mother's and fathers' highest level of education attained: (1) elementary school graduate and below (2) junior high school graduate, (3) senior high school graduate, (4) university graduate.

### Statistical analysis

The TAALS study team assigned weights to weight variables in order to reduce bias caused by survey design and differences between respondents and non-respondents. We employed sample weights to obtain unbiased estimators (e.g., mean and standard error) and minimize the discrepancy between the distribution structure of the sample and that of the population (28). Participants missing depressive symptoms records were being removed (Supplementary File 3). We used chi-square tests for categorical variables to analyze the characteristics of participants, depressive symptoms, and clustering of unhealthy behaviors by sex and school type. Generalized linear mixed models were used to assess the association between binary clustering of unhealthy behaviors and depressive symptoms. Models stratified by sex and school type were employed to determine the association between categorical clustering of unhealthy behaviors and depressive symptoms in each sub-population. Generalized linear mixed models were adjusted for BMI, unhealthy behaviors and socioeconomic determinants. We used SPSS software (version 25; IBM SPSS Statistic) to conduct all statistical analyses. A p-value of 0.05 indicates statistical significance.

## Results

Overall, among the 18,509 participants, 8,964 (48.4%) were male, and 9,545 (51.6%) were female. In addition, 6,846 (37.0%) participants were junior high school students, 4,783 (25.8%) were high school students, and 6,880 (37.2%) were vocational high school students.

### Proportion of depressive symptoms and clustering of unhealthy behaviors

Table 1 shows the proportion of critical variables and the variations across sex and school type. Almost one–third of participants had depressive symptoms (31.4%). As for unhealthy behaviors, over three-fourths of participants were IPA (77.7%). Those who engaged in FSSBC and SBSB were dominant (60.2 and 54.3%, respectively). Over two-thirds of participants exhibited clustering of unhealthy behaviors (68.5%). When stratified by sex, females were more likely than males to report depressive symptoms (34.7 vs. 28.0%, respectively; *p* < 0.001). Females were also more likely than males to participate in IPA (85.7 vs. 69.1%, respectively; *p* < 0.001). However, male participated in FSSBC (64.7 vs. 56.0%, respectively; *p* < 0.001) and SBSB (55.5 vs. 53.4%, respectively; *p* = 0.002) more than females. There was no significant difference between males and females in terms of clustering of unhealthy behaviors (67.9 vs. 69.2%, respectively; *p* = 0.057). When stratified by school type, it was shown that vocational high school students were more likely to report depressive symptoms than those from senior high school and junior high school (35.6 vs. 34.0% vs. 25.4%, respectively; *p* < 0.001). Vocational high school students were the most likely ones among three school types to participate in IPA, SBSB, and clustering of unhealthy behaviors (Supplementary File 4).

**Table 1:** Characteristics of participants.

**Variables**	**Total (*n* = 18,509)**	**Sex**			**School type**			
		**Male (*****n*** = **8,964)**	**Female (*****n*** = **9,545)**	***p***	**Junior (*****n*** = **6,846)**	**Senior (*****n*** = **4,783)**	**Vocational (*****n*** = **6,880)**	***p***
Depressive symptom, *n* (%)				<0.001				<0.001
Yes	5,816 (31.4)	2,506 (28)	3,310 (34.7)		1,742 (25.4)	1,624 (34)	2,450 (35.6)	
No	12,693 (68.6)	6,458 (72)	6,235 (65.3)		5,104 (74.6)	3,159 (66)	4,430 (64.4)	
Sex, *n* (%)								<0.001
Male					3,350 (48.9)	2,054 (42.9)	3,560 (51.7)	
Female					3,496 (51.1)	2,729 (57.1)	3,320 (48.3)	
Age, *n* (%)				0.151				
16 years old	11,663 (63)	5,614 (62.6)	6,049 (63.4)			4,783 (41)	6,880 (59)	
13 years old	6,846 (37)	3,350 (37.4)	3,496 (36.6)		6,846 (100)			
School type, *n* (%)				<0.001				
Senior	4,783 (25.8)	2,054 (22.9)	2,729 (28.6)					
Junior	6,846 (37)	3,350 (37.4)	3,496 (36.6)					
Vocational	6,880 (37.2)	3,560 (39.7)	3,320 (34.8)					
Body mass index, *n* (%)				<0.001				<0.001
Underweight	5,380 (29.6)	2,548 (29)	2,832 (30.3)		2,670 (40)	1,091 (23)	1,619 (24)	
Normal	9,648 (53.1)	4,345 (49.4)	5,303 (56.7)		3,023 (45.3)	2,875 (60.7)	3,750 (55.6)	
Overweight or obese	3,125 (17.2)	1,901 (21.6)	1,224 (13.1)		977 (14.6)	773 (16.3)	1,375 (20.4)	
FSSBC, *n* (%)				<0.001				<0.001
≥3 times/week	11,125 (60.2)	5,792 (64.7)	5,333 (56)		3,853 (56.5)	2,906 (60.8)	4,366 (63.5)	
0–2 times/week	7,353 (39.8)	3,156 (35.3)	4,197 (44)		2,968 (43.5)	1,875 (39.2)	2,510 (36.5)	
IPA, *n* (%)				<0.001				<0.001
Yes	9,819 (53.3)	3,707 (41.6)	6,112 (64.3)		2,834 (41.6)	2,843 (59.6)	4,142 (60.6)	
No	8,600 (46.7)	5,200 (58.4)	3,400 (35.7)		3,978 (58.4)	1,926 (40.4)	2,696 (39.4)	
SBSB, *n* (%)				0.002				<0.001
Yes	10,047 (54.4)	4,960 (55.5)	5,087 (53.4)		2,933 (43)	2,182 (45.7)	4,932 (71.8)	
No	8,427 (45.6)	3,980 (44.5)	4,447 (46.6)		3,894 (57)	2,597 (54.3)	1,936 (28.2)	
Clustering of behaviors, *n* (%)				<0.001				<0.001
≥2 behaviors	10,776 (58.7)	5,010 (56.5)	5,766 (60.8)		3,129 (46.2)	2,733 (57.4)	4,914 (72)	
0–1 behavior	7,584 (41.3)	3,862 (43.5)	3,722 (39.2)		3,644 (53.8)	2,031 (42.6)	1,909 (28)	

### Association between clustering of unhealthy behaviors and depressive symptoms

Table 2 shows the results of generalized linear mixed models with binary clustering of unhealthy behaviors and after adjustment for BMI, other unhealthy behaviors (adjusted model 1), and social determinants (adjusted models 2) (Supplementary File 5). In the unadjusted model, depressive symptoms were more likely to be found among participants who exhibited clustering of unhealthy behaviors (cOR = 1.81, 95% CI: 1.73–7.90). This association became slightly decreased after adjusted for other unhealthy behaviors (adjusted model 1, aOR = 1.58, 95% CI: 1.52–1.65) and further decreased after adjusted for socioeconomic determinants (adjusted model 2, aOR = 1.53, 95% CI: 1.48–1.58).

**Table 2:** Association with depressive symptoms across clustering of unhealthy behaviors, sex and school type.

**Variables**	Crude model^*^		Adjusted model I^**^		Adjusted model II^***^	
	**OR (95% CI)**	***p***	**OR (95% CI)**	***p***	**OR (95% CI)**	***p***
**Clustering of unhealthy behaviors**						
≥2 behaviors	1.81 (1.73–1.90)	< 0.001	1.58 (1.52–1.65)	< 0.001	1.53 (1.48–1.58)	< 0.001
0–1 behavior	Ref		Ref		Ref	
**Sex**						
Male	0.74 (0.71–0.76)	< 0.001	0.72 (0.70–0.74)	< 0.001	0.66 (0.64–0.67)	< 0.001
Female	Ref		Ref		Ref	
**School type**						
Senior	0.97 (0.92–1.03)	0.324	1.03 (1.00–1.06)	0.041	1.10 (1.09–1.11)	< 0.001
Junior	0.69 (0.64–0.72)	< 0.001	0.77 (0.73–0.81)	< 0.001	0.7 (0.67–0.73)	< 0.001
Vocational	Ref		Ref		Ref	

* Adjusted for BMI.

** Adjusted for BMI, Binge drinking, Smoking, Skipping Breakfast, Emotional Eating, Eating while doing something, Nutrition label reading.

*** Adjusted for BMI, Binge drinking, Smoking, Skipping Breakfast, Emotional Eating, Eating while doing something, Nutrition label reading, Bullying Experience, Peer support, School support, and Parental Education.

#### Generalized linear mixed models stratified by sex

The associations between categorical clustering of unhealthy behaviors and depressive symptoms stratified by sex were shown in Table 3. Overall, depressive symptoms were most likely to occur among the triple unhealthy behavior subgroup (aOR = 2.27, 95% CI: 2.13–2.42). Among the dual unhealthy behavior subgroup, IPA combined SBSB subgroup (OR = 1.93, 95% CI: 1.81–2.01) was more likely than IPA combined FSSBC subgroup (OR = 1.82, 95% CI: 1.70–1.94) and SBSB combined FSSBC subgroup (OR = 1.85, 95% CI: 1.66–2.05) to report depressive symptoms. All single unhealthy behavior significantly associated with depressive symptoms, in detail, IPA (OR = 1.45, 95% CI: 1.37–1.54), SBSB (OR = 1.36, 95% CI: 1.27–1.46), FSSBC (OR = 1.35, 95% CI: 1.24–1.47).

**Table 3:** Generalized linear mixed models demonstrated the association between clustering of unhealthy behaivors and depressive symptoms stratified by sex.

**Variables**	**All**		**Female**		**Male**	
	**OR (95% CI)**	***p***	**OR (95% CI)**	***p***	**OR (95% CI)**	***p***
**Clustering of unhealthy behaviors** ^*^						
FSSBC^**^	1.35 (1.24–1.47)	<0.001	1.77 (1.62–1.95)	<0.001	1.10 (0.99–1.22)	0.066
SBSB^**^	1.36 (1.27–1.46)	<0.001	1.46 (1.41–1.51)	<0.001	1.35 (1.19–1.53)	<0.001
IPA^**^	1.45 (1.37–1.54)	<0.001	1.61 (1.27–2.03)	<0.001	1.32 (1.11–1.66)	0.002
FSSBC+SBSB	1.85 (1.66–2.05)	<0.001	2.14 (1.91–2.41)	<0.001	1.63 (1.35–1.96)	<0.001
IPA + FSSBC	1.82 (1.70–1.94)	<0.001	1.87 (1.67–2.10)	<0.001	1.86 (1.76–1.97)	<0.001
IPA+SBSB	1.93 (1.81–2.01)	<0.001	2.12 (2.05–2.19)	<0.001	1.77 (1.55–2.04)	<0.001
All unhealthy behaviors	2.27 (2.13–2.42)	<0.001	2.38 (2.25–2.51)	<0.001	2.19 (1.86–2.57)	<0.001
No unhealthy behavior	Ref		Ref		Ref	

* Adjusted for school type, BMI, Binge drinking, Smoking, Skipping Breakfast, Emotional Eating, Eating while doing something, Nutrition label reading, Bullying Experience, Peer support, School support, and Parental Education.

** IPA, Insufficient Physical Activity; SBSB, Screen based sedentary behaviors; FSSBC, Frequent sugar-sweetened beverage consumption.

## Discussion

This study investigates the association between the clustering of unhealthy behaviors and depressive symptoms among Taiwanese adolescents in 2015. Our findings highlighted three significant predictors of depressive symptoms along with a complex pattern of health behaviors, individual factors, and social determinants. Our research findings contributed to the current knowledge of health consequences of unhealthy behaviors among adolescents (3).

Similar to previous studies (22, 25, 26), our main findings indicated that depressive symptoms were common in Taiwanese adolescents. During 2015–2017, another nationwide surveillance of DSM-5 mental disorders also highlighted the high prevalence rates of overall mental disorders among Taiwanese children (aged 10–13 years) (22). These rates were higher than other previous analyses from the Project for the Health of Adolescents (aged 13–18 years) in Southern Taiwan which was conducted in 2004 (25). In addition, these results might be considerably higher than the global estimation of mental health burden among adolescent (41). Our study and various other studies on mental health matters in Taiwan (22, 26) raise the issue of the importance of preventing depression and suicide among Taiwanese children and adolescents.

We found that clustering of unhealthy behaviors was positively associated with depressive symptoms. Another study also indicated the positive association between physical activity and depressive symptoms (14). Furthermore, other studies suggest to consider physical activity as a practical approach to improve mental health among children and adolescents (24, 42). There was additional evidence of the dose-response relationship between screen-based sedentary behavior and depression (15, 35). A variety of previous studies highlighted that overconsumption of sugar-sweetened beverages was a risk factor for depressive symptoms (23, 43). Diet, physical activity and sedentary behavior often referred to as “energy balance-related behaviors” were frequently considered as the critical risk factors for overweight and obese (12, 44). Meanwhile, our study addressed the correlation of diet, physical activity and sedentary behavior on mental health conditions in adolescents, which were rarely assessed in previous studies (45).

From children and adolescent mental health standpoint, risk factors for depressive symptoms are frequently related to psychological and social factors such as cognition, interpersonal skills, low self-esteem, self-emotion regulation (16, 46). Recent evidence based on RCT study results demonstrated the efficacy of physical activity intervention for improving depressive symptoms (42). However, larger RCT studies targeting whole adolescent populations rarely assessed outcomes of physical activity, sedentary behavior and diet interventions on mental health (47). Therefore, assessment on depressive symptoms should be integrated into interventions on improving energy-balance related behaviors such as diet, physical activity and sedentary behaviors among children and adolescents.

Consistent with previous studies in other countries (8, 18), we found that multiple unhealthy behaviors were commonly co-existed among Taiwanese adolescents. A 5-years follow-up cohort study in Australia concluded lifestyle risk behaviors are prevalent among young adults, and that risk behaviors concurrent with another one (45). Another study consisted of nationally representative U.S children and adolescents indicated excessive screen time and poor diet were commonly co-occurred. The multiple unhealthy behaviors were observed to be increased with age (31). A systematic review on clustering of unhealthy behaviors among children and adolescents demonstrated insufficient physical activity and sedentary behavior cluster were frequently observed, and that the cluster relationship was influenced by age, sex and socio-economic status (12).

In Taiwan, there were a few studies investigating the clustering of unhealthy behaviors among adults. For instance, a prospective cohort study demonstrated the positive association between unhealthy behaviors and all-cause mortality (48). Our study contributed to one of the first evidence that clustering of unhealthy behaviors is positively associated with depression among adolescents in Taiwan. Therefore, our study findings may have several implications on policy. For instance, our results suggest that the prevention interventions should target multiple behaviors at the same time for a better outcome. The outcomes of reducing IPA, SBSB, FSSBC are not only improving obesity but also mental health condition among adolescents. Prevention approaches that address multiple behaviors may be more cost-effective (17). On the other hand, mental health prevention should be integrated into the Health Promotion School Program (49), especially for senior high schools and vocational high schools.

This study has several methodological limitations. First, because this is a cross-sectional study, the findings cannot be used to indicate the causal effect between clustering of unhealthy behaviors and depressive symptoms. Second, based on the questionnaire responses, measurements of both behaviors and depressive symptoms may contain information bias. Participants' responses may be influenced by their ability to recall events in the past, and by their social desirability (31). Third, some factors significantly related to depressive symptoms, for instance, self-emotion regulation (47), were not adjusted in our analysis. A prior meta-analytic review highlighted the negative association between self-regulation and depressive symptom, obesity, substance abuse among young adolescents (50). Finally, this analysis was not able to explain the relationship pattern of depressive symptoms, emotional eating and FSSBC. Previous studies demonstrated emotional eating as a significant mediator between depression, anxiety and weight gain (51). Among Taiwanese adolescents, the association between emotional eating and FSSBC has been indicated elsewhere (36).

Our research has several strengths. The nationwide sampling offered a representative and consistent sample of Taiwan's adolescent population. Furthermore, our data revealed a variety of unhealthy behaviors among vocational high school students, senior high school students, and junior high school students, including IPA, SBSB and FSSBC as well as adolescent mental health issue. Adolescent demographics showed diverse lifestyles, social determinants, and unhealthy behaviors including smoking and binge drinking when stratified by school types, all of which likely to be significant driven factors of an adolescent's depressive symptoms. The stratified results enable the development of customized health promotion strategies for each subgroup. The critical variables associated with depressive symptoms have been controlled in our adjusted model.

## Conclusion

Our study findings show that depressive symptoms are common in Taiwanese adolescents and positively associated with unhealthy behaviors in a complex behavioral pattern. Therefore, conducting an assessment on risk behavior clustering is essential for directing the development of mental health prevention strategies because it provides insights into which risk behaviors should be targeted together. Furthermore, it becomes crucial to develop school-based health promotion interventions tailored to different subgroups for better intervention results.

## Data availability statement

The raw data supporting the conclusions of this article will be made available by the authors, without undue reservation.

## Ethics statement

The studies involving human participants were reviewed and approved by Taipei Medical University—Joint Institutional Review Board (TMU—JIRB-201410043). Written informed consent to participate in this study was provided by the participants' legal guardian/next of kin.

## Author contributions

CB, Y-WC, and H-YC: conceptualization. CB, L-YL, and H-YC: methodology. L-YL, Y-WC, and H-YC: validation, supervision, and writing—review and editing. CB and C-JL: formal analysis and investigation. H-YC: resources. CB and H-YC: data curation. CB and L-YL: writing original draft preparation. All authors have read and agreed to the published version of the manuscript.
