# Supplementary material for: Association between clustering of unhealthy behaviors and depressive symptom among adolescents in Taiwan: A nationwide cross-sectional survey
Source: Front Public Health. 2023 Mar 9;11:1049836. doi: 10.3389/fpubh.2023.1049836 (PMC10035074; doi:10.3389/fpubh.2023.1049836)
Supplement: Supplementary file 2 [file Table_2.DOCX]

**Supplemetary file 2**.

**Eight categories based on unhealthy behaviors**

| **Category** | **Descriptions** |
| --- | --- |
| No Unhealthy behaviors (ref) | An individual, during the last week, were:  FSSBC < 3 times  SBSB < 2 hours per day  Physically active at least 60 mins on 5 days or more |
| FSSBC | An individual, during the last week, were:  FSSBC ≥3 times  SBSB < 2 hours per day  Physically active at least 60 mins on 5 days or more |
| SBSB | An individual, during the last week, were:  FSSBC < 3 times  SBSB ≥ 2 hours per day  Physically active at least 60 mins on 5 days or more |
| IPA | An individual, during the last week, were:  FSSBC < 3 times  SBSB < 2 hours per day  Physically active at least 60 mins on less than 5 days |
| FSSBC+SBSB | An individual, during the last week, were:  FSSBC ≥ 3 times  SBSB ≥ 2 hours per day  Physically active at least 60 mins on 5 days or more |
| IPA + FSSBC | An individual, during the last week, were:  FSSBC ≥ 3 times  SBSB < 2 hours per day  Physically active at least 60 mins on less than 5 days |
| IPA+SBSB | An individual, during the last week, were:  FSSBC < 3 times  SBSB ≥ 2 hours per day  Physically active at least 60 mins on less than 5 days |
| All unhealthy behaviors | An individual, during the last week, were:  FSSBC ≥ 3 times  SBSB ≥ 2 hours per day  Physically active at least 60 mins on less than 5 days |

IPA: Insufficient Physical Activity; SBSB: Screen based sedentary behaviors; FSSBC: Frequent sugar-sweetened beverage consumption con
