# Supplementary material for: Association between clustering of unhealthy behaviors and depressive symptom among adolescents in Taiwan: A nationwide cross-sectional survey
Source: Front Public Health. 2023 Mar 9;11:1049836. doi: 10.3389/fpubh.2023.1049836 (PMC10035074; doi:10.3389/fpubh.2023.1049836)
Supplement: Supplementary file 3 [file Table_3.DOCX]

Additional File 2

Proportion of Missing data (N=18509)

| **Variables** | **Missing data** | Proportion |
| --- | --- | --- |
|  |  |  |
| **Clustering of unhealthy behaviors** |  |  |
|  | 149 | 0.81% |
|  |  |  |
| **Sex** |  |  |
|  | 0 | 0 |
|  |  |  |
| **School type** | 0 | 0 |
| **Insufficient physical activity** | 90 | 0.49% |
| **Screen time based sedentary behavior** | 35 | 0.19% |
| **Sugar sweetened bevarages frequently consumption** | 31 | 0.17% |
|  |  |  |
|  |  |  |
| **Binge drinking** | 11 | 0.06% |
|  |  |  |
|  |  |  |
| **Smoking** | 7 | 0.04% |
|  |  |  |
|  |  |  |
| **Skipping Breakfast** | 8 | 0.04% |
|  |  |  |
|  |  |  |
| **Emotional Eating** | 87 | 0.47% |
|  |  |  |
|  |  |  |
| **Eating while doing something** | 40 | 0.22% |
|  |  |  |
|  |  |  |
| **Nutrition label reading** | 64 | 0.35% |
|  |  |  |
|  |  |  |
| **Bullying Experience** | 54 | 0.29% |
|  |  |  |
|  |  |  |
| **Peer support** | 112 | 0.61% |
|  |  |  |
|  |  |  |
| **School support** | 380 | 2.05% |
|  |  |  |
|  |  |  |
