# Supplementary material for: Association between clustering of unhealthy behaviors and depressive symptom among adolescents in Taiwan: A nationwide cross-sectional survey
Source: Front Public Health. 2023 Mar 9;11:1049836. doi: 10.3389/fpubh.2023.1049836 (PMC10035074; doi:10.3389/fpubh.2023.1049836)
Supplement: Supplementary file 4 [file Table_4.DOCX]

Supplementary file 4

Characteristics of participants

| **Variables** | **Total (n = 18,509)** | **Male (n = 8,964)** | **Female (n = 9,545)** | ***p*** |
| --- | --- | --- | --- | --- |
| **Age, *n* (%)** |  |  |  | 0.151 |
| 16 years old | 11,663 (63) | 5614 (62.6) | 6049 (63.4) |  |
| 13 years old | 6846 (37) | 3350 (37.4) | 3496 (36.6) |  |
| **School type, *n* (%)** |  |  |  | <0.001 |
| Senior | 4783 (25.8) | 2054 (22.9) | 2729 (28.6) |  |
| Junior | 6846 (37) | 3350 (37.4) | 3496 (36.6) |  |
| Vocational | 6880 (37.2) | 3560 (39.7) | 3320 (34.8) |  |
| **Body mass index, *n* (%)** |  |  |  | <0.001 |
| <18.5 | 5380 (29.6) | 2548 (29) | 2832 (30.3) |  |
| ≥24 | 3125 (17.2) | 1901 (21.6) | 1224 (13.1) |  |
| 18.5-24 | 9648 (53.1) | 4345 (49.4) | 5303 (56.7) |  |
| **Depressive symtoms, *n* (%)** |  |  |  | <0.001 |
| Yes | 5816 (31.4) | 2506 (28) | 3310 (34.7) |  |
| No | 12,693 (68.6) | 6458 (72) | 6235 (65.3) |  |
| **Fast food consumption, *n* (%)** |  |  |  | <0.001 |
| ≥3 times/week | 2949 (16) | 1718 (19.2) | 1231 (12.9) |  |
| 0–2 times/week | 15,504 (84) | 7215 (80.8) | 8289 (87.1) |  |
| **High fat snack consumption, *n* (%)** |  |  |  | <0.001 |
| ≥3 times/week | 4897 (26.5) | 2583 (28.9) | 2314 (24.3) |  |
| 0–2 times/week | 13,574 (73.5) | 6360 (71.1) | 7214 (75.7) |  |
| **Processed meat product consumption, *n* (%)** |  |  |  | <0.001 |
| ≥3 times/week | 5512 (29.9) | 3184 (35.6) | 2328 (24.4) |  |
| 0–2 times/week | 12,951 (70.1) | 5756 (64.4) | 7195 (75.6) |  |
| **Dessert food consumption, *n* (%)** |  |  |  | <0.001 |
| ≥3 times/week | 8611 (46.6) | 3879 (43.4) | 4732 (49.7) |  |
| 0–2 times/week | 9859 (53.4) | 5064 (56.6) | 4795 (50.3) |  |
| **SSBs consumption, *n* (%)** |  |  |  | <0.001 |
| ≥3 times/week | 11,125 (60.2) | 5792 (64.7) | 5333 (56) |  |
| 0–2 times/week | 7353 (39.8) | 3156 (35.3) | 4197 (44) |  |
| **Insufficient physical activity, *n* (%)** |  |  |  | <0.001 |
| Yes | 14312 (77.7) | 6157 (69.1) | 8155 (85.7) |  |
| No | 4107 (22.3) | 2750 (30.9) | 1357 (14.3) |  |
| **Screen time-based sedentary behaviors, *n* (%)** |  |  |  | 0.002 |
| Yes | 10,047 (54.4) | 4960 (55.5) | 5087 (53.4) |  |
| No | 8427 (45.6) | 3980 (44.5) | 4447 (46.6) |  |
| **Clustering of unhealthy behaviors, *n* (%)** |  |  |  | 0.057 |
| Yes | 12584 (68.5) | 6021 (67.9) | 6563 (69.2) |  |
| No | 5776 (31.5) | 2851 (32.1) | 2925 (30.8) |  |
| **Emotional Eating, *n* (%)** |  |  |  | <0.001 |
| Yes | 5861 (31.8) | 2654 (29.8) | 3207 (33.7) |  |
| No | 12,561 (68.2) | 6264 (70.2) | 6297 (66.3) |  |
| **Skipping Breakfast, *n* (%)** |  |  |  | <0.001 |
| Yes | 2067 (11.2) | 918 (10.2) | 1149 (12) |  |
| No | 16,434 (88.8) | 8041 (89.8) | 8393 (88) |  |
| **Eating while doing something, *n* (%)** |  |  |  | 0.166 |
| Yes | 9690 (52.5) | 4656 (52.1) | 5034 (52.8) |  |
| No | 8779 (47.5) | 4282 (47.9) | 4497 (47.2) |  |
| **Nutrition label reading, *n* (%)** |  |  |  | <0.001 |
| Yes | 8641 (46.8) | 4019 (45) | 4622 (48.6) |  |
| No | 9804 (53.2) | 4913 (55) | 4891 (51.4) |  |
| **Binge drinking, *n* (%)** |  |  |  | <0.001 |
| Yes | 1186 (6.4) | 735 (8.2) | 451 (4.7) |  |
| No | 17,312 (93.6) | 8223 (91.8) | 9089 (95.3) |  |
| **Smoking, *n* (%)** |  |  |  | <0.001 |
| Yes | 1030 (5.6) | 780 (8.7) | 250 (2.6) |  |
| No | 17,472 (94.4) | 8177 (91.3) | 9295 (97.4) |  |
| **Being bullied experience, *n* (%)** |  |  |  | 0.002 |
| Yes | 6456 (35) | 3224 (36.1) | 3232 (33.9) |  |
| No | 11,999 (65) | 5702 (63.9) | 6297 (66.1) |  |
| **Peer support, *n* (%)** |  |  |  | <0.001 |
| Yes | 15,841 (86.1) | 7433 (83.6) | 8408 (88.5) |  |
| No | 2556 (13.9) | 1463 (16.4) | 1093 (11.5) |  |
| **School support, *n* (%)** |  |  |  | <0.001 |
| Yes | 16,905 (93.2) | 7963 (90.8) | 8942 (95.5) |  |
| No | 1224 (6.8) | 804 (9.2) | 420 (4.5) |  |
| **Father Education, *n* (%)** |  |  |  | 0.790 |
| University graduate | 5953 (35.6) | 2861 (35.5) | 3092 (35.6) |  |
| Senior high school graduate | 7156 (42.7) | 3458 (43) | 3698 (42.6) |  |
| Junior high school graduate | 3078 (18.4) | 1476 (18.3) | 1602 (18.4) |  |
| Elementary school graduate and below | 553 (3.3) | 255 (3.2) | 298 (3.4) |  |
| **Mother Education, *n* (%)** |  |  |  | 0.118 |
| University graduate | 5722 (34.1) | 2769 (34.7) | 2953 (33.5) |  |
| Senior high school graduate | 8213 (48.9) | 3885 (48.7) | 4328 (49.2) |  |
| Junior high school graduate | 2169 (12.9) | 991 (12.4) | 1178 (13.4) |  |
| Elementary school graduate and below | 685 (4.1) | 339 (4.2) | 346 (3.9) |  |
