# Supplementary material for: Association between clustering of unhealthy behaviors and depressive symptom among adolescents in Taiwan: A nationwide cross-sectional survey
Source: Front Public Health. 2023 Mar 9;11:1049836. doi: 10.3389/fpubh.2023.1049836 (PMC10035074; doi:10.3389/fpubh.2023.1049836)
Supplement: Supplementary file 5 [file Table_5.DOCX]

Supplementary file 5 - Adjusted models

Association of clustering of unhealthy behaviors and covariates with exhibiting depressive symptoms

| **Variables** | **Crude model*** |  | **Adjusted model I**** |  | **Adjusted model II***** |  |
| --- | --- | --- | --- | --- | --- | --- |
|  | OR (95% CI) | *p* | OR (95% CI) | *p* | OR (95% CI) | *p* |
| **Clustering of unhealthy behaviors** |  |  |  |  |  |  |
| ≥2 behaviors | 1.81 (1.73-1.90) | <0.001 | 1.58 (1.52-1.65) | <0.001 | 1.53 (1.48-1.58) | <0.001 |
| 0-1 behavior | Ref |  | Ref |  | Ref |  |
| **Sex** |  |  |  |  |  |  |
| Male | 0.74 (0.71-0.76) | <0.001 | 0.72 (0.70-0.74) | <0.001 | 0.66 (0.64-0.67) | <0.001 |
| Female | Ref |  | Ref |  | Ref |  |
| **School type** |  |  |  |  |  |  |
| Senior | 0.97 (0.92-1.03) | 0.324 | 1.03 (1.00-1.06) | 0.041 | 1.10 (1.09-1.11) | <0.001 |
| Junior | 0.69 (0.64-0.72) | <0.001 | 0.77 (0.73-0.81) | <0.001 | 0.7 (0.67-0.73) | <0.001 |
| Vocational | Ref |  | Ref |  | Ref |  |
| **BMI** |  |  |  |  |  |  |
| <18.5 | 0.99 (0.97-1.02) | 0.051 | 0.97 (0.93-1.00) | 0.051 | 0.98 (0.95-1.01) | 0.107 |
| ≥ 24 | 1.04 (0.97-1.10) | 0.059 | 1.08 (0.99-1.16) | 0.059 | 1.02 (0.96-1.09) | 0.453 |
| 18.5-24 | Ref |  | Ref |  | Ref |  |
| **Binge drinking** |  |  |  |  |  |  |
| Yes |  |  | 1.57 (1.40-1.76) | <0.001 | 1.48 (1.31-1.68) | <0.001 |
| No | N/A |  | Ref |  | Ref |  |
| **Smoking** |  |  |  |  |  |  |
| Yes |  |  | 1.27 (1.13-1.42) | 0.001 | 1.19 (1.17-1.21) | <0.001 |
| No | N/A |  | Ref |  | Ref |  |
| **Skipping Breakfast** |  |  |  |  |  |  |
| Yes |  |  | 1.99 (1.85-2.14) | <0.001 | 1.84 (1.69-1.99) | <0.001 |
| No | N/A |  | Ref |  | Ref |  |
| **Emotional Eating** |  |  |  |  |  |  |
| Yes |  |  | 1.52 (1.46-1.58) | <0.001 | 1.44 (1.38-1.50) | <0.001 |
| No | N/A |  | Ref |  | Ref |  |
| **Eating while doing something** |  |  |  |  |  |  |
| Yes |  |  | 1.32 (1.24-1.41) | <0.001 | 1.35 (1.27-1.43) | <0.001 |
| No | N/A |  | Ref |  | Ref |  |
| **Nutrition label reading** |  |  |  |  |  |  |
| Yes |  |  | 0.89 (0.81-0.98) | 0.024 | 0.94 (0.85-1.03) | 0.184 |
| No | N/A |  | Ref |  | Ref |  |
| **Bullying Experience** |  |  |  |  |  |  |
| Yes |  |  |  |  | 1.97 (1.79-2.18) | <0.001 |
| No | N/A |  | N/A |  | Ref |  |
| **Peer support** |  |  |  |  |  |  |
| Yes |  |  |  |  | 0.35 (0.34-0.37) | <0.001 |
| No | N/A |  | N/A |  | Ref |  |
| **School support** |  |  |  |  |  |  |
| Yes |  |  |  |  | 0.53 (0.47-0.6) | <0.001 |
| No | N/A |  | N/A |  | Ref |  |
| **Father Education** |  |  |  |  |  |  |
| University graduate |  |  |  |  | 1 (0.8-1.25) | 0.983 |
| Senior high school graduate |  |  |  |  | 0.96 (0.78-1.19) | 0.567 |
| Junior high school graduate |  |  |  |  | 0.93 (0.74-1.15) | 0.306 |
| Elementary school graduate and below | N/A |  | N/A |  | Ref |  |
| **Mother Education** |  |  |  |  |  |  |
| University graduate |  |  |  |  | 1 (0.81-1.24) | 0.753 |
| Senior high school graduate |  |  |  |  | 0.99 (0.81-1.2) | 0.644 |
| Junior high school graduate |  |  |  |  | 1.1 (0.89-1.36) | 0.420 |
| Elementary school graduate and below | N/A |  | N/A |  | Ref |  |

* Adjusted for BMI

** Adjusted for BMI, Binge drinking, Smoking, Skipping Breakfast, Emotional Eating, Eating while doing something, Nutrition label reading

*** Adjusted for BMI, Binge drinking, Smoking, Skipping Breakfast, Emotional Eating, Eating while doing something, Nutrition label reading, Bullying Experience, Peer support, School support, and Parental Education
